# Human *Babesia odocoilei* and *Bartonella* spp. co-infections in the Americas

**DOI:** 10.1186/s13071-024-06385-4

**Published:** 2024-07-11

**Authors:** Ricardo G. Maggi, Ana Cláudia Calchi, Charlotte O. Moore, Emily Kingston, Edward B. Breitschwerdt

**Affiliations:** 1 College of Veterinary Medicine, North Carolina State University, Intracellular Pathogens Research Laboratory Comparative Medicine Institute, Raleigh, NC USA; 2 Department of Pathology, Reproduction and One Health, Vector-Borne Bioagents Laboratory (VBBL), School of Agricultural and Veterinarian Sciences (FCAV) - São Paulo State University (UNESP), Jaboticabal, SP Brazil

**Keywords:** *Bartonella*, *Babesiosis*, *Babesia odocoilei*, Zoonotic diseases, Co-infection, Fatigue, Neurology

## Abstract

**Background:**

In recent years, *Babesia* and *Bartonella* species co-infections in patients with chronic, nonspecific illnesses have continued to challenge and change the collective medical understanding of “individual pathogen” vector-borne infectious disease dynamics, pathogenesis and epidemiology. The objective of this case series is to provide additional molecular documentation of *Babesia odocoilei* infection in humans in the Americas and to emphasize the potential for co-infection with a *Bartonella* species.

**Methods:**

The development of improved and more sensitive molecular diagnostic techniques, as confirmatory methods to assess active infection, has provided increasing clarity to the healthcare community.

**Results:**

Using a combination of different molecular diagnostic approaches, infection with *Babesia odocoilei* was confirmed in seven people suffering chronic non-specific symptoms, of whom six were co-infected with one or more *Bartonella* species.

**Conclusions:**

We conclude that infection with *Babesia odocoilei* is more frequent than previously documented and can occur in association with co-infection with *Bartonella* spp.

**Graphical Abstract:**

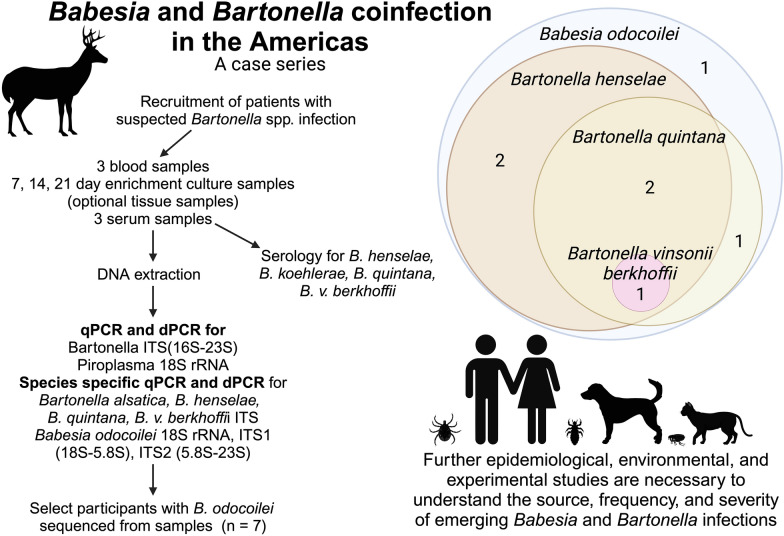

## Background

Human babesiosis, an emerging zoonosis caused by apicomplexan protozoa of the genus *Babesia*, has been described on nearly every continent [1, 2]. Worldwide to date, at least nine different species have been reported to cause infections in human beings: *Babesia bigemina*, *B. crassa* (and *B. crassa*-like), *B. divergens, B. duncani*, *B. microti*, *B. motasi, B. odocoilei* and *B. venatorum* [3–47]. In the USA, the main *Babesia* species that infect humans are *B. microti*, *B. duncani* and *B. divergens*-like [2, 48–50]. Transmission occurs mainly by tick bite, but there are reports of transmission by transfusion of contaminated blood, organ transplantation and transplacental transmission [51–54]. In addition to asymptomatic infection, babesiosis can be associated with non-specific symptoms or severe, life-threatening hemolytic anemia, the severity of which can be related to immunosuppressive factors, such as splenectomy [55].

In Canada and the US, *Babesia odocoilei*, a protozoal species mostly associated with infection in cervids (white tailed deer, elk, reindeer and moose) and musk ox, has also been reported to infect people [47]. A closely related species (in many cases reported as *B. odocoilei*) has also been reported in red deer from Austria, the Czech Republic, England and Germany [56, 57]. *Ixodes scapularis* and *Dermacentor variabilis* are considered the primary tick vectors for transmission of *B. odocoilei* in the USA and Canada [58–66], whereas *I. ricinus* and *I. canisuga* have been associated with transmission of *B. odocoilei*-like organisms in Europe [56, 57].

In 2003, Herwaldt and colleagues [47] first reported the possibility of *B. odocoilei* infection in two human patients (one from Italy and one from Austria), both experiencing night sweats, chills, fevers, profound fatigue, increased thirst, muscle aches and sleep disturbances, symptoms historically associated with babesiosis. Both patients were seroreactive to *B. divergens* and seronegative to *B. microti* antigens*. Babesia* DNA sequences from both patients were identical and were phylogenetic related to *B. odocoilei*, a parasite of white-tailed deer in North America. In retrospect, due to limitations associated with species discrimination using the 18S rRNA gene, these individuals were likely infected with *B. divergens* rather than *B. odocoilei*, as these two closely related ruminant *Babesia* form a sister phylogenetic clade [47]. In 2021, Scott and colleagues [39] reported *B. odocoilei* infection in two humans, both experiencing night sweats, chills, fevers, profound fatigue, increased thirst, muscle aches and sleep disturbances. The 18S rRNA DNA sequences from these two individuals were most similar to *B. odocoilei* sequences obtained from ticks and cervids in Canada.

Based primarily on serological test results, most often associated with *B. duncani* as the test antigen, babesiosis has often been diagnosed as a co-infection in patients with Lyme disease, caused by *Borrelia burgdorferi,* a spirochete also transmitted by *I. scapularis.* The primary vector for transmission of *B. duncani* is *Ixodes pacificus*, a tick species localized to the west coast of North America. To the authors’ knowledge *B. duncani* DNA has never been amplified from a tick, pet dog (frequently tested by PCR diagnostically), human or wild animal east of the Rocky Mountains. Thus, a discrepancy has existed between human serology and vector epidemiology results, which may have been associated with serological cross-reactivity between *B. duncani* and *B. odocoilei*, as reported by Scott and colleagues [39].

Despite clinical and epidemiological support for vector transmission, whether or the extent to which *Bartonella* spp., and in particular *Bartonella henselae,* are transmitted by ticks in North America remains undetermined [67–72]. Although the common “cat flea” *Ctenocephalides felis* is the most important vector for *B. henselae* transmission worldwide, other vectors including woodlouse hunter spiders, rat mites and ants (Australia and the US) have been implicated as a source of *B. henselae* vector transmission to humans [70, 73–75]. In addition to needle stick transmission to a veterinarian, *B. henselae* DNA has been amplified from dolphins, Beluga whales, and sea otters in the marine environment and mongoose in the Caribbean islands [8, 9, 76–81]. Although vectors are the primary modes for *B. henselae* transmission, viability of this bacteria within aquatic, marine and terrestrial environments may represent an underestimated source for human infections. Currently, the medical importance of the genus *Bartonella* remains underappreciated and incompletely studied [38]. An important area of emerging research focuses on the potential role of *B. henselae* as a cause or cofactor in patients with psychoses, schizophrenia and other neuropsychiatric presentations, which makes defining mode(s) of transmission, duration of infection and the medical consequences of chronic infection of the utmost importance [13, 82–88].

The advent of more sensitive molecular diagnostic techniques continues to change the collective medical understanding of vector-borne infectious disease dynamics, pathogenesis and epidemiology, with important but incompletely understood implications for patients. Droplet digital PCR assays (ddPCRs) were developed and validated in our laboratory to enhance the sensitivity of detection of *Babesia, Bartonella* and *Borrelia* spp. DNA in animal and human patient specimens [89, 90]. The enhanced sensitivity of ddPCR facilitated the detection of *B. odocoilei* DNA in the seven research participants, six of whom were co-infected with one or more *Bartonella* spp. Additional molecular validation allowed for confirmation of *B. odocoilei* infection, an emerging human pathogen. The objective of this case series is to provide additional molecular documentation of *B. odocoilei* infection in humans in the Americas and to emphasize the potential for co-infection with a *Bartonella* species.

## Methods

With the cooperation of their attending physician, blood and serum were submitted by all participants. Tissues (two individuals) and an intravenous port sample (another individual) were submitted by three participants. All samples were processed in the Intracellular Pathogens Research Laboratory (IPRL), College of Veterinary Medicine, North Carolina State University, for attempted isolation or molecular detection of a *Bartonella* species. All study participants provided three blood and serum specimens collected within a 7-day period. These individuals were tested because of a history of arthropod or animal contact as a component of an Institutional Review Board (IRB) approved study entitled "Detection of Bartonella Species in the Blood of People with Extensive Animal Contact" (North Carolina State University Institutional Review Board, IRB#s 4925-03 and 164-08-05). Permission to test for other infectious agents was individually granted. A standardized questionnaire including age, gender, animal and arthropod exposure, outdoor activity, travel, clinical symptoms, duration of illness and comorbid conditions was completed by each individual or by the parents of the two children. The duration of illness varied substantially among individuals, as did prior diagnostic evaluations and previous treatments.

As described previously [87, 91, 92], each participant was tested using five indirect fluorescent antibody (IFA) assays, each representing a unique *Bartonella* species or genotype. *Bartonella vinsonii* subsp. *berkhoffii* (genotypes I and II), *B. henselae* (strain San Antonio 2), *B. koehlerae* and *B. quintana* IgG antibodies were determined using DH82 cell culture-grown bacteria as antigens and following standard IFA techniques with fluorescein conjugated goat anti-human IgG. A sample was considered *Bartonella* sp. seroreactive if an IFA titer of ≥ 1:64 was obtained for any one or more antigen.

**Fig. 1:**
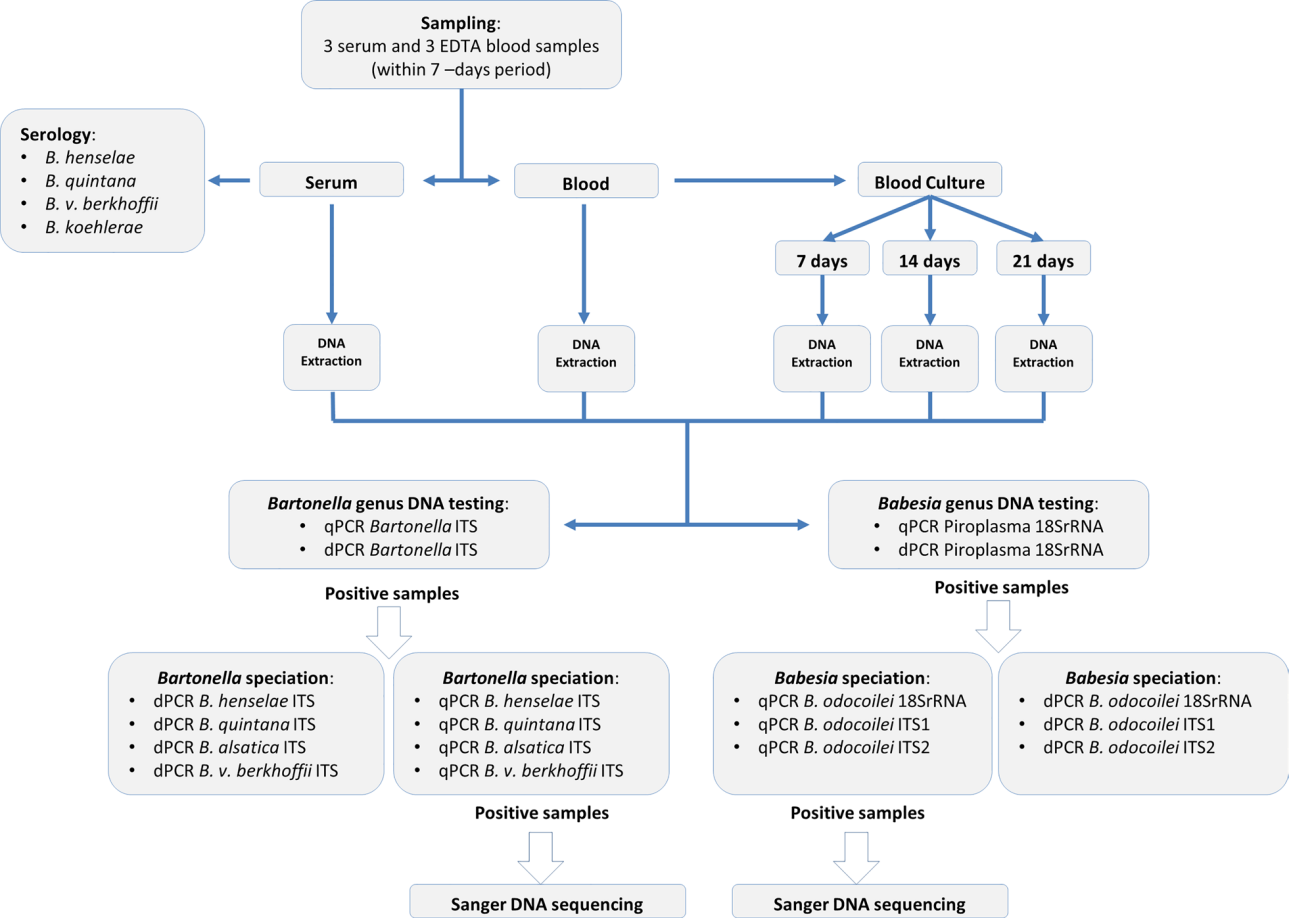
Enrichment culture and molecular testing approach used in this study [87, 89, 113]

The culture and molecular testing approach used in this study is depicted in Fig. 1 [93]. Following DNA extraction from blood, serum, tissues and an intravenous port sample, amplification of the human hydroxymethylbilane synthase gene was used as the housekeeping human reference gene. The *Bartonella* spp. intergenic spacer 16S-23S rRNA (ITS) region and *Babesia* 18S rRNA gene were targeted by quantitative PCR (qPCR, CFXOpus thermocycler, Bio-Rad, Hercules, CA), ddPCR (QX One Droplet Digital PCR, Bio-Rad, Hercules, CA) and dPCR (QIAcuity 5 plex digital PCR, QIAgen Qiagen, Valencia, CA) using primers and probes as previously described [89, 90, 94]. Blood, serum and enrichment blood cultures incubated in Brugge liquid culture media for 7, 14 and 21 days were tested by qPCR and ddPCR targeting the Bartonella 16S-23 S intergenic spacer (ITS) region and the *Babesia* 18S rRNA gene [89, 90]. DNA was manually extracted (Qiagen DNeasy Blood and Tissue Kit, Qiagen, Valencia, CA, USA) following the manufacturer’s tissue extraction protocol from omental carcinomatosis, uterine wall, fibroid tissues and the intravenous port sample for qPCR and ddPCR testing. A sample was considered PCR positive if qPCR or ddPCR generated a positive result [90]. *Babesia* 18S rRNA qPCR ddPCR-positive DNA extractions were subsequently tested using two *B. odocoilei*-specific ITS probes that were developed to confirm the identity of the *Babesia* species. Sequences were aligned and compared with GenBank sequences using AlignX software (Vector NTI Suite 6.0, InforMax, Inc.).

## Results

Demographic data for the seven individuals infected with *B. odocoilei* are summarized in Table 1. Ages ranged from 2.5 to 62 years old. Six individuals were female. Four individuals were veterinary workers, two were students, one of whom was a veterinarian’s daughter, and one was a pre-school-age child. They resided in four USA states and Mexico. Reported illness duration at the time of testing ranged from days to 14 years. Symptoms reported by individuals on the study questionnaire are listed in Table 2. Fatigue was the most frequently reported symptom, followed by memory loss, headache, irritability/rage/aggression and poor appetite. Night sweats and air hunger, symptoms historically associated with babesiosis, were not respondent options on the questionnaire. Nearly all seven study participants reported exposure to the same arthropod and insect vectors, including fleas, ticks, biting flies, mosquitoes and spiders. No study participant reported exposure to bedbugs, and only participant 4, co-infected with *Bartonella henselae* and *B. quintana*, reported louse exposure, although her mother (participant 3) was also co-infected with these same two *Bartonella* spp. Based upon the medical history, questionnaire responses, prolonged duration of illness or failure to observe a tick for study participant 7, it was not possible to determine the mode or timing of pathogen transmission.

**Table 1:** Demographic data reported for the six study participants at the time of research blood and tissue specimen submission in 2022

Case	Age (years)	Gender	Occupation	Residence	Duration of illness
1	62	F	Veterinary technician/dog trainer	New Jersey, USA	6 years
2	10.6	M	Student	New Jersey, USA	5.5 years
3	57	F	Veterinarian	Mexico City, Mexico	6 years
4	22	F	Student, veterinarian technician	Mexico City Mexico	13 years
5	53	F	Veterinary technician	North Carolina, USA	14 years
6	46	F	Veterinarian	Michigan, USA	3 years
7	2.5	F	NA	Oklahoma, USA	days to 2 years*

^*^Acute onset rash that resolved after several days. Subsequent symptoms reported in the medical history persisted for 2 years

**Table 2:** Symptoms reported by the seven individuals or their parents on the study questionnaire

Symptoms	Case1	Case2	Case3	Case4	Case5	Case6	Case7
Fatigue	x		x	x	x	x	
Difficulty remembering	x	x	x		x		
Disoriented (confused by time or place)		x	x		x		
Irritability/rage/aggression		x		x	x		
Eye pain					x		
Difficulty sleeping (insomnia)		x	x	x		x	
Chronic fatigue			x	x			
Bladder dysfunction			x	x	x		
Poor appetite		x		x	x		
Vomiting				x			
Weight gain			x	x		x	
Tachycardia (rapid heart rate)			x	x			
Tremors or shaking				x			
Headache	x		x	x	x		
Mental confusion (disordered thoughts)			x	x	x		
Hallucinations				x			
Blurred vision					x		
Sleepiness			x	x		x	
Balance problems					x		
Bowel dysfunction						x	x
Shortness of breath			x	x			
Weight loss					x		
Diarrhea							
Depression			x	x	x		
Rash/skin lesion			x	x			x
Anxiety/panic attacks		x		x			
Muscle weakness		x					
Muscle pain	Arms						
Loss of sensation/ numbness					Fingers	Right hand	
Joint pain	Fingers, wrist, knees					Right knee/hip	
Other symptoms			Desire to shake or strike arms and legs		Dry mouth, change of taste	Pelvic pain/Bloating, thyroid cyst	

All seven participants were initially determined to be 18S rRNA piroplasma dPCR positive. Infection was confirmed to be a *Babesia *species based upon DNA sequencing using qPCR amplified DNA. Subsequent DNA sequence comparisons, based on amplification of the ITS1 and/or ITS2 regions, were generated to confirm infection with *B. odocoilei*. Table 3 provides *B. odocoilei* 18S rRNA, ITS1 and ITS2 region sequence similarities for blood, enrichment blood culture, tissues and a surgically removed intravenous port for the seven participants compared to *B. odocoilei* sequences from elk, white-tailed-deer, caribou and reindeer deposited in GenBank.

**Table 3:** *Babesia* ITS1 and ITS2 DNA sequence identity for seven people infected with *Babesia odocoilei*. Note: N/A not amplified

Case	Sample	ITS-1		ITS-2	
		% identity	GenBank #	% identity	GenBank #
1	20670 C7	104/105 (99.1%) *B. odocoilei* AY339748	PP550653	169/169 (100%) *B. odocoilei* AY345122	PP550644
		104/105 (99.1%) *B. odocoilei* AY339747			
	20672 C7	104/105 (99.1%) *B. odocoilei* AY339747	PP550654	169/169 (100%) *B. odocoilei* AY345122	PP550645
		104/105 (99.1%) *B. odocoilei* AY339754			
2	20696	Not sequenced NSS*		Not sequenced	
3	20453	104/105 (99.1%) *B. odocoilei* AY339747	PP550655	169/169 (100%) *B. odocoilei* AY345122	PP550646
4	20820 C21	Not sequenced		N/A	
	20822 C7	104/105 (99.1%) *B. odocoilei* AY339747	PP550656	168/169 (99.4%) *B. odocoilei* AY339758	PP550647
		104/105 (99.1%) *B. odocoilei* AY339754			
5	20461	104/105 (99.1%) *B. odocoilei* AY339747	PP550657	169/169 (100%) *B. odocoilei* AY345122	PP550648
	20464	Not sequenced		169/169 (100%) *B. odocoilei* AY339758	PP550649
6	20510	104/105 (99.1%) *B. odocoilei* AY339747	PP550658	167/169 (99%) *B. odocoilei* AY339758	PP550650
	20524	104/105 (99.1%) *B. odocoilei* AY339747	PP550659	N/A	
		104/105 (99.1%) *B. odocoilei* AY339754			
7	20666 C14	104/105 (99.1%) *B. odocoilei* AY339747	PP550660	169/169 (100%) *B. odocoilei* AY345122	PP550651
		104/105 (99.1%) *B. odocoilei* AY339754			
	20806 C14	104/105 (99.1%) *B. odocoilei* AY339747	PP550661	169/169 (100%) *B. odocoilei* AY339758	PP550652
		104/105 (99.1%) *B. odocoilei* AY339754			
	21,219 C14	103/105 (98.1%) *B. odocoilei* AY339747	PP592351		

^*^Not successfully sequenced

Six of seven individuals were seroreactive to one or more *Bartonella* spp. antigens (Table 4). *Bartonella henselae* DNA was amplified and sequenced from three individuals, whereas infection was detected by *B. henselae* species-specific probes in two additional participants (Table 4). *Bartonella quintana* DNA was amplified and sequenced from one individual, whereas infection was detected in three additional participants by *B. quintana* species-specific probes. Participant 2 was co-infected with *B. henselae, B. vinsonii* subsp. *berkhoffii* and *B. quintana.* A veterinarian residing in northern Michigan was seronegative to four of five *Bartonella* spp. antigens, and *Bartonella* DNA was not amplified from her blood, serum, enrichment blood cultures or tissues. The 2.5-year-old child was *Bartonella* spp. seronegative at acute illness onset; however, the child seroconverted to all five *Bartonella* spp. antigens after administration of antibiotics (Table 4).

**Table 4:** *Bartonella* species indirect fluorescent antibody titers and *Bartonella* spp. designation as determined by DNA sequencing or species-specific probes

Case	Bvb TI	Bvb TII	Bh SA2	Bk	Bq	*Bartonella sp.*
1	1:64*	1:32*	1:128*	1:16*	1:32*	*B. henselae*
2	1:128	1:128	1:128	1:128	1:64	*B. quintana/B. henselae/B. v. berkhoffii*
3	1:64	1:64	1:256	1:128	1:256	*B. quintana/B. henselae*
4	1:64	1:64	1:64	1:128	1:64	*B. quintana/B. henselae*
5	1:256	1:32	1:256	1:128	1:16	*B. henselae*
6	< 1:16	1:16	1:16	1:32	1:64	Not detected
7	< 1:16	< 1:16	< 1:16	< 1:16	< 1:16	*B. quintana/B. henselae*

^*^Each patient provided three blood specimens for research testing during 2022. Serological testing was performed using five indirect fluorescent antibody assays. Reciprocal antibody titers were obtained for *B. vinsonii* subsp. *berkhoffii* strain I (BvbI), *B. vinsonii* subsp. *berkhoffii* strain II (BvbII), *B. henselae* strain *San Antonio* 2 (Bh SA2), *B. koehlerae* (Bk) and *B. quintana* (Bq)

An abbreviated summary of each participants medical history follows:

Participant 1 was diagnosed with peritoneal carcinomatosis 3 months prior to study entry. She had been diagnosed with bartonellosis 5 years earlier and was treated with various oral antibiotic combinations between 2017 and 2022. Despite treatment interventions, she reported progressive fatigue, memory loss, headaches and joint/muscle pain spanning 6 years in duration. Co-infection with *B. odocoilei* (ITS1 and ITS2 primers) and *B. henselae* (ITS region) were confirmed by PCR amplification and DNA sequencing from blood or enrichment blood cultures. Neither *Bartonella* nor *Babesia* DNA was amplified from omental carcinomatosis tissue obtained 3 weeks after study entry.

Participant 2 developed neuropsychiatric symptoms consistent with Pediatric Acute Onset Neuropsychiatric Syndrome. Until 4 years old, this boy was an extremely verbal, high-functioning child, with normal physical, psychological and academic development. Acutely, he experienced a rapid regression in speech, involving both receptive and expressive language. He also developed acute anxiety, severe deterioration in sleep patterns and additional changes in behavior and personality. According to his father, “the boy’s condition deteriorated to the point where he was almost entirely non-verbal, physically weak, and impaired, and consumed by anxiety that significantly impeded his well-being.” He was treated with clonazepam and clonidine for several years. At the time of study entry, “undiagnosed brain disease of 5 years and 10 months duration” was listed as a working diagnosis, despite having been examined by 12 physicians in various specialties. *Babesia odocoilei* DNA was amplified using 18S rRNA, ITS1 and ITS2 primers from one of three blood samples, whereas *B. henselae*, *B. quintana* and *B. vinsonii* subsp. *berkhoffii* were detected in three, two and one samples, respectively, using *Bartonella* dPCR speciation probes.

Participant 3 reported a history of severe depression spanning many years. She had been evaluated by psychiatrists in Mexico, including the National Institute of Psychiatrists TAC, RM (Instituto Nacional de Psiquiatría Ramon de la Fuente Muñiz in Mexico City, Calzada Mexico-Xochimilco 101, Tlalpan). She had been refractory to a spectrum of psychiatric drugs, participated in a pharmacogenetic study, received neurostimulator therapy and received two cycles of intravenous ketamine. She had previously declined electroconvulsive therapy. As a companion animal veterinarian, she never recalled being bitten by a tick but had experienced flea bites and cat scratches. Other than travel to the Southeastern US for professional meetings, she had never traveled outside of Mexico. At the time of initial research testing, she was medically disabled and reported progressively worsening symptoms. She was co-infected with *B. henselae, B. quintana* and *B. odocoilei*. She was treated for bartonellosis with rifampicin and doxycycline for 2 months, after which doxycycline, 100 mg twice daily, was continued for 8 months, during which time she reported symptom resolution and improved memory and was able to stop psychiatric medications. Within months of stopping antibiotics, symptoms, including depression, anxiety, suicidal ideation and memory loss, returned. *Bartonella quintana* and *B. henselae* DNA was amplified and sequenced from her blood post-antibiotic treatment. As *B. odocoilei* infection was only retrospectively confirmed by DNA amplification of the 18S rRNA, ITS1 and ITS2 in 2024 in blood samples obtained 5 months apart, she had not been treated for babesiosis.

Participant 4 (daughter of participant 3) reported a combination of symptoms suggestive of autonomic nervous system dysfunction and neuropsychiatric illness, including suicidal/homicidal thoughts. At 9 years of age, she was diagnosed with oppositional defiant disorder (ODD) because of anxiety, severe depression, headaches, nightmares and hallucinations. She subsequently attempted suicide and over the ensuing years developed tachycardia, repeated urinary infections, homicidal thoughts and cutaneous stretch marks. She had assisted her mother, a veterinarian, in her clinic caring for animal patients for at least 2 years prior to illness onset. On the questionnaire, she reported cat and dog bites in the same year in which she was diagnosed with ODD. Except for a visit to the southwestern US, she had never traveled outside of Mexico. When her January 2022 samples were retrospectively tested, only *B. odocoilei* DNA was amplified and sequenced (ITS1 and ITS2 regions); however, her September 2022 samples contained both *B. odocoilei* (as determined by DNA sequences) and* B. quintana* and * B. henselae* (determined using species-specific probes) DNA. The mother and daughter were both infected with *B. odocoilei, B. quintana* and *B. henselae.*

Participant 5 first entered our IRB-approved study in 2011 at 42 years of age; she reported a 24- year duration of illness. As a veterinary technician, she sought research testing due to a 3-month history of medically refractory migraines, including non-responsiveness to three consecutive daily injections of intravenous dihydroergotamine. Occupationally, and personally, she had frequent contact with animals and arthropod vectors. She had an extensive travel history within the US and had visited Central America and South Africa. She reported bites and scratches from numerous animal species. She was seroreactive to *B. henselae* San Antonio 2 (SA2) strain type (1:256) but not seroreactive to *B. henselae* Houston 1, (IFA titer < 1:16) or the four other *Bartonella* spp. or genotypes. *Bartonella henselae* SA2 strain DNA was amplified and sequenced from two blood samples collected 4 days apart. When treated for bartonellosis, her migraines resolved. Within 3 months, she seroreverted (*B. henselae* SA2 IFA titer < 1:16) and was qPCR negative from blood and BAPGM enrichment blood culture DNA extractions. Due to persistence of other symptoms listed in Table 2, she was retested on multiple occasions. *Bartonella henselae* DNA was amplified from her blood in 2013, 2016 and 2017. Despite treatment with multiple oral and intravenous antibiotics for prolonged durations for bartonellosis, her health continued to deteriorate to the point that she became medically disabled. Between 2019 and 2020, *Bartonella* spp. ddPCR was positive in blood or in a 14- or 21-day enrichment blood culture. Again, in January 2022, *B. henselae* DNA was amplified and sequenced from her blood sample. One week later her catheter port was removed. *Babesia odocoilei* DNA was independently amplified and sequenced from the port and from the catheter, presumably containing biofilm.

Participant 6, a very physically active veterinarian, reported mild, progressive fatigue and insomnia of 3-year duration prior to research testing. Due to endometriosis and uterine fibroids, a hysterectomy was performed 2 weeks after blood and serum were submitted for *Bartonella* testing. She was only seroreactive to *B. quintana. Bartonella* spp. DNA was not amplified from blood, serum or enrichment blood cultures. *Babesia odocoilei* DNA was amplified and sequenced from her uterus (the highest *B. odocoilei* probe result for any study participant) and from one fibroid.

**Fig. 2:**
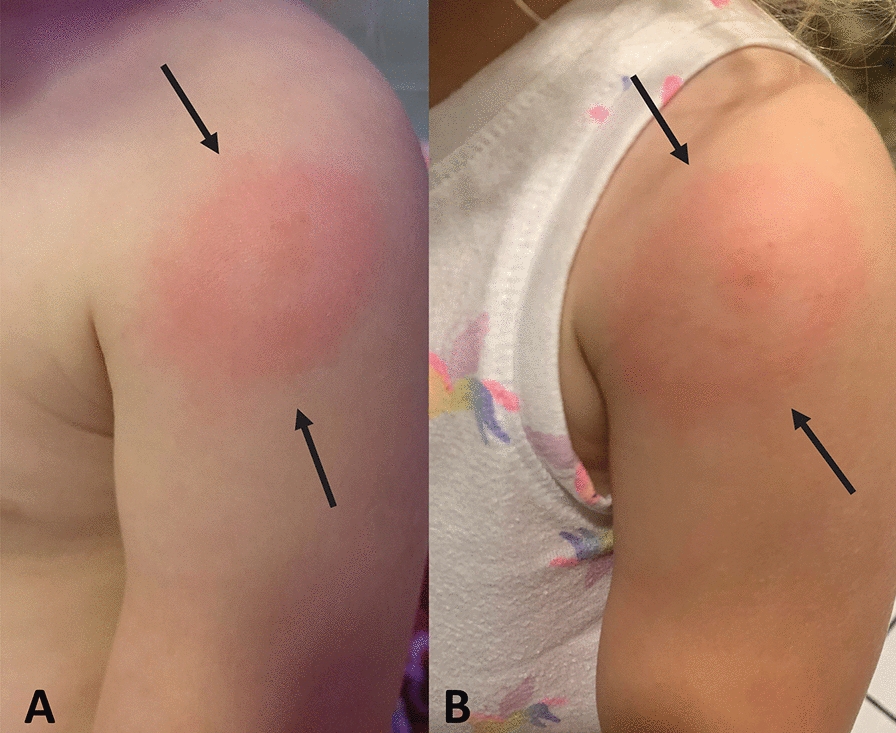
Photographs taken by the parents of a rapid onset, homogeneous, non-pruritic red rash on the upper left arm (arrows) in a 2.5-year-old girl (study participant 7) co-infected with *Babesia odocoilei*, *Bartonella henselae* and *Bartonella quintana*. **A** Image obtained at 9:30 p.m., June 6, 2022, when the rash was first noted. **B** Image obtained at 2 p.m., June 7, 2022, roughly 15 h after the rash was first visualized, illustrating an expansive lesion and bullseye-like appearance. Written permission was granted for publication of the photographs

Participant 7 developed a “bulls eye” rash (Fig. 2) in June 2022 after playing that day in her parents' backyard in Oklahoma. A local pediatrician reported to the parents that she had seen several other children in the city with similar rashes around that period. No tick was attached or observed. Over the ensuing months, the child developed night sweats, knee pain, nightmares and sleep apnea. Despite prior antibiotic treatment, in November 2023, her father reported the following: “She has experienced an array of moderate symptoms over the last few years. She has significant night sweats, frequently complains of knee pain, nightmares, and appears to have sleep apnea at times during the night. There are other symptoms but those are the most common. I'd consider her healthy now, but I have seen some minor nightmares and one night of night sweats in the last few weeks.” Research testing results generated for blood specimens collected between June 2022 and January 2024 are summarized in Table 5. Both she and her father (2024, data not shown) were infected with *B. quintana* and *B. odocoilei* with identical ITS1 DNA sequences. Although other participants were piroplasm 18S rRNA dPCR + at multiple testing time points, chronic *B. odocoilei* infection was confirmed based on DNA sequencing of the 18S rRNA, ITS1 and ITS2 regions in this girl and participant 3.

**Table 5:** Sequential *Bartonella* spp. indirect fluorescent antibody titers and *Bartonella* and *Babesia odocoilei* infection, as determined by DNA sequencing (^) or genus/species-specific probes (^^), in a 2.5-year-old girl. Each patient provided three blood specimens for research testing during 2022. Serological testing was performed using five indirect fluorescent antibody assays. Reciprocal antibody titers were obtained for *Bartonella vinsonii *subsp. *berkhoffii* strain I (BvbI), *B. vinsonii* subsp. *berkhoffii* strain II (BvbII), *B. henselae* strain San Antonio 2 (Bh SA2), *B. koehlerae* (Bk) and *B. quintana* (Bq)

Case date	Bvb TI	BvbII	Bh SA2	Bk	Bq	*Bartonella* species	*Babesia* species
6/14/2022	< 1:16	< 1:16	< 1:16	< 1:16	1:32	*Bartonella* sp.*^^*	*B. odocoilei*^
8/30/2022	1:32	1:16	1:64	1:32	1:64	*Bh^^, Bq^^*	*B. odocoilei*^^
2/6/2023	1:64	1:128	1:64	1:64	1:64	*Bartonella* spp.*^^*	Positive
1/29/2024	1:64	1:32	1:32	1:16	1:64	*Bartonella* sp.*^^*	Positive

**Fig. 3:**
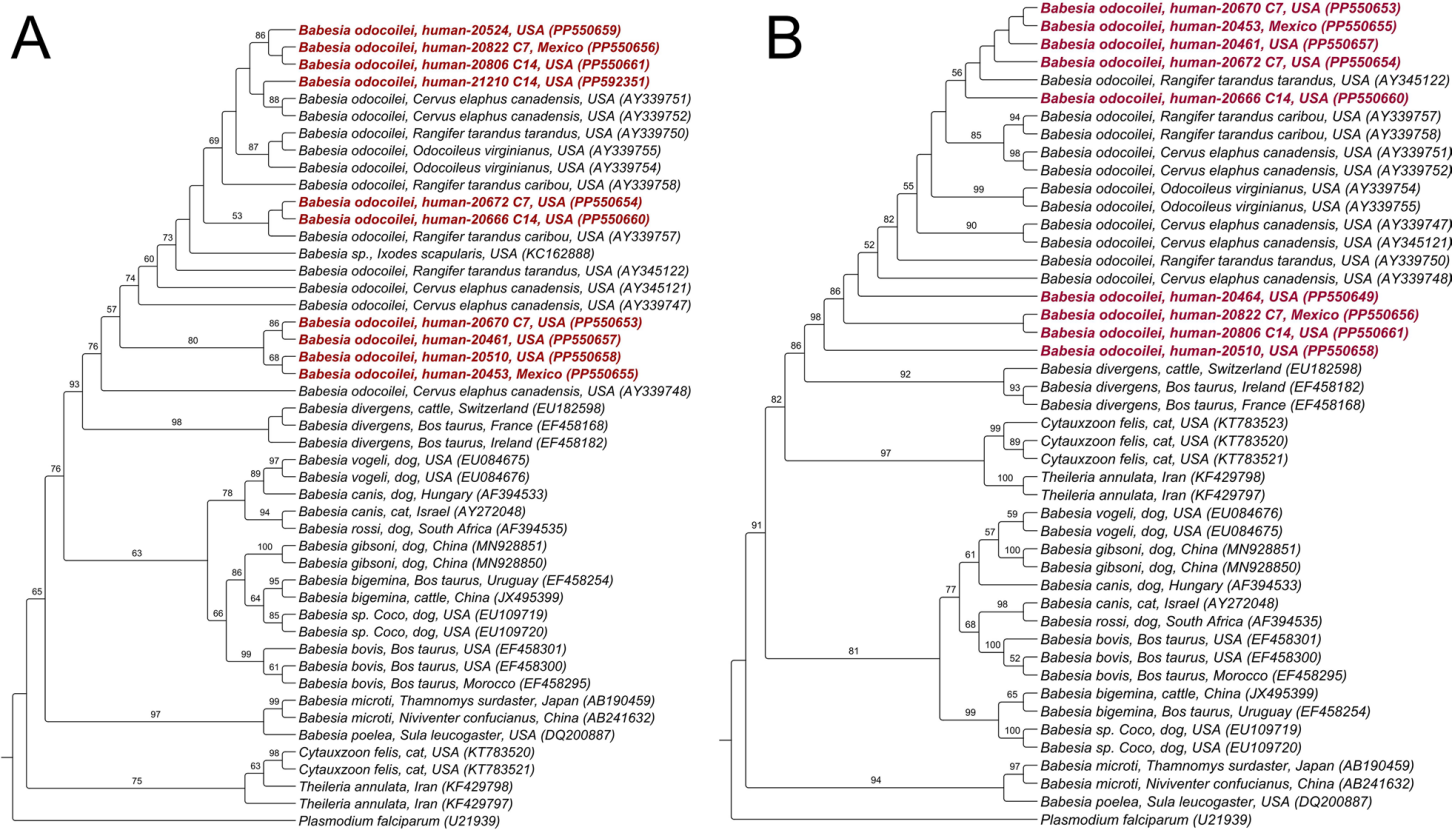
Phylogenetic tree of *Babesia* ITS1 (**A**) and ITS2 (**B**) sequences using HKY + G evolutionary model. The sequences were aligned with other homologous sequences of each gene retrieved from the database (GenBank) using the MAFFT software [95] and edited via Bioedit v. 7.0.5.3 [96]. W-IQ-Tree software was used for choosing the evolutionary model following AIC criterion as well as for phylogenetic analysis inferred the Maximum Likelihood method (available online: http://iqtree.cibiv.univie.ac.at/) [97]. Clade support indices were evaluated through bootstrap analyses of 1000 repetitions. The phylogenetic trees were edited using Treegraph 2.0.56–381 beta software [98]

The datasets generated and analyzed during the current study are available in GenBank and can be accessed through accession numbers PP550637–PP550643; PP592351, PP592352 and PP550653–PP550661; and PP550644–PP550652 for 18S rRNA gene, ITS-1 and ITS-2, respectively. A phylogenetic tree using ITS1 and ITS2 region sequences, compared with closely related species and genera, are depicted in Fig. 3a and b, respectively.

## Discussion

For over 3 decades, our research has primarily focused on the genus *Bartonella*. The seven individuals in this study were initially tested for *Bartonella* sp. infection, which was documented in all but one person. Because co-infection with *Babesia, Bartonella* and *Borrelia* species has been increasingly reported or suspected in patients with chronic, therapeutically recalcitrant medical symptoms, we developed and validated a multiplex ddPCR assay to amplify DNA of all three genera [89]. Previously, co-infections with these three genera have usually been diagnosed on the basis of serology [99–103]. Indirect diagnostic methods such as serology can only provide evidence for pathogen exposure, can lack specificity due to potential cross-reactivity with other microorganisms or other species within the same genus and can lack sensitivity in immunocompromised patients or when infection is accompanied by a state of immunological anergy [104–106]. Due to low level bacteremia and the relapsing nature of *Bartonella* spp. infections, standard diagnostic methods, such as blood smear examination and PCR (conventional or quantitative/real time) using DNA extracted from blood, have relatively low direct detection sensitivities. Due to these limitations, we used an enrichment culture approach in conjunction with obtaining three blood collections within a 7-day period to enhance the direct detection sensitivity of qPCR and ddPCR for a molecular diagnosis of bartonellosis. Based upon the preliminary results derived from these seven individuals, similar diagnostic sensitivity challenges are to be anticipated when attempting to achieve molecular confirmation of *B. odocoilei* infections in patient blood or serum specimens. It is important to emphasize that three blood and serum specimens were collected from study participants within a 7-day period and that blood, serum and three enrichment blood cultures incubated in Brugge liquid culture media for 7, 14 and 21 days were tested by qPCR and ddPCR targeting the *Bartonella* ITS region. Thus, 15 independent DNA extractions from blood serum and enrichment cultures were required to generate the *Bartonella* spp. PCR results reported in this study. For each participant, most DNA extractions were PCR negative. Also in this study, qPCR lacked sensitivity, as 16 samples (55.2%, 16/29) were piroplasm 18S rRNA gene dPCR positive, whereas all were qPCR negative. Only two (6.9%) samples were piroplasm 18S rRNA qPCR positive and dPCR negative. Similarly, despite extracting DNA from blood, serum and 7-, 14- and 21-day enrichment triple-draw blood cultures (in total 15 independent DNA extractions/participant), only one to three samples were *Babesia* dPCR positive per participant, and in some instances only 1–2 dots were amplified, reflecting an extremely low *B. odocoilei* parasitemia. If enrichment culture had not been employed in the testing strategy, *B. odocoilei* parasitemia would not have been confirmed in participants 1, 4 and 7. Collectively, these results emphasize the inherent challenges in documenting infection with both blood-borne pathogens in patient blood specimens.

*Babesia odocoilei* species-specific 18S rRNA, ITS-1 and ITS-2 qPCR and dPCR assays developed in this study proved to be reasonably concordant. All seven *B. odocoilei* 18S rRNA dPCR samples were ITS qPCR positive. Specifically, eight of 11 dPCR-positive samples for *B. odocoilei* ITS1 region were qPCR positive for the same target gene and seven of the 10 positive samples for the *B. odocoilei* ITS2 region were qPCR positive. As only 13/41 (31.7%) samples were positive for the specific *B. odocoilei* assays (either as 18S rRNA, ITS1 and/or ITS2), the difference may be due to concurrent co-infection with another *Babesia* species (unpublished data). It is also possible that the low parasitemia in these samples prevented detection using these *B. odocoilei*-specific PCR assays. Thus, obtaining definitive molecular confirmation of *B. odocoilei* infection in a non-reservoir host remains challenging, despite the enhanced sensitivity of dPCR.

Although blood transfusion transmission of *Babesia* spp. is a public health concern, ticks are considered the primary if not the sole vector for transmission, including *B. odocoilei.* However, there is substantial evidence of dog-to-dog *Babesia gibsoni* transmission via fighting. Bites, particularly bites from American Staffordshire (Pitbull) terriers, are a mode of transmission [107]. As five individuals in this study were veterinary workers, including the daughter of a veterinarian with similar animal exposures, and all reported ownership or exposure to dogs, zoonotic transmission could not be ruled out. The child in this study developed a circumscribed bullseye-like rash after playing in the parent’s yard during a particularly rainy summer period in Oklahoma. An insect bite was suspected, as an attached tick was not seen. The skin lesion was distinctly different from mosquito bites that the child had experienced previously. In addition to a tick bite, it is perhaps prudent to consider other modes of *B. odocoilei* transmission in future studies.

Although it is increasingly clear that *Babesia* and *Bartonella* species can induce longstanding blood-borne intraerythrocytic infections in immunocompetent human patients, the extent to which chronic infection contributes to immune system dysfunction, autoimmune phenomena and non-specific symptoms, such as severe fatigue, is substantially less clear. Thus, the symptoms reported by these individuals cannot be solely or partially attributed to these infections. Participants 1 and 5, diagnosed with bartonellosis years earlier, remained *B. henselae* seroreactive, and *B. henselae* DNA was amplified from their blood, despite long-term treatment with multiple antibiotic combinations administered following diagnosis. Participant 3 remained infected with *B. quintana* post-antibiotic treatment. Based upon duration of illness, participants 1–5 were chronically ill, with non-specific symptoms, as reported previously in bartonellosis patients [108–110]. In contrast, the child had an acute onset rash followed by symptoms potentially consistent with babesiosis (night sweats) and bartonellosis (knee pain, nightmares). As participant 6 was a veterinarian, failure to document infection with a *Bartonella* spp. in blood, serum or tissues was unexpected as was the amplification of *B. odocoilei* DNA from her uterus and one uterine fibroid. Three independent PCR targets (18S rRNA gene, ITS1 and ITS2), performed at different time points, were positive for her uterine tissue, making DNA carryover or laboratory contamination unlikely. Notably, *B. odocoilei* DNA was not amplified from her blood, serum or enrichment blood cultures (15 independent DNA extractions), suggesting the possibility of *Babesia* localization to her reproductive tissues. Experimental transplacental transmission of *B. microti* has been reported using rats as has natural transplacental transmission of *Babesia bovis* to cattle, *B. caballi* to horses, *B. gibsoni* to dogs, *B. microti* to mice (*Peromyscus leucopus*) and cases of suspected *B. microti* transmission to children [3, 4, 111]. Whether or the extent to which *B. odocoilei* infection may have contributed to her endometriosis or uterine fibroids is unknown. Endometriosis, a disease of unknown causation, does have immunological features that are reported in association with chronic infections [112]. Also, *Babesia* and *Bartonella* transplacental transmission from mother to daughter might be a consideration for participants 3 and 4.

In the context of limitations, it was not possible to determine when or by what mode of transmission individuals in this study were infected with *B. odocoilei* or a *Bartonella* species. As the molecular detection of both organisms in patient blood specimens is difficult to achieve, considerable time, effort and testing were performed on multiple samples at different times to obtain an adequate quantity of DNA for successful sequencing. Some participants had been treated for babesiosis, bartonellosis or both infections prior to and after our initial 18S rRNA *Babesia* focused testing began in 2022. Also, we did not anticipate potential growth of *B. odocoilei* in enrichment culture, which clearly deserves future research consideration.

## Conclusions

Considering the relatively short 1-year (2022) study period with subsequent follow-up for some participants, the wide geographic distribution of study participants, the variable and often non-specific symptoms, and the fact that *B. odocoilei* DNA was present in blood, tissues, biofilm and a port, we conclude that infection with this *Babesia* sp. is more prevalent than previously suspected. Also, in contrast to acute babesiosis, which is most often associated with an acute hemolytic anemia or thrombocytopenia, these hematological abnormalities were not reported by study participants, potentially further limiting a physician’s decision to test for babesiosis. The findings reported in this study clearly justify additional applied research to define the medical importance of human *B. odocoilei* and *Bartonella* spp. co-infections in Mexico, the USA, and potentially elsewhere.

## Data Availability

Sequences analyzed during the current study are available in GenBank under the accession numbers: PP550637-PP550643; PP592351, PP592352, and PP550653-PP550661; PP550644-PP550652 for 18S rRNA gene, ITS-1 and ITS-2, respectively.
